# Evaluating SegFormer and U-Net for Real-Time Sensor-Based Sorting: From Semantic Segmentation to Sorting

**DOI:** 10.3390/s26217040

**Published:** 2026-11-07

**Authors:** Lukas Roming, Felix Lehnerer, Jürgen Hock, Kevin Liang, Josua Ehret, Tridib Ghosh, Georg Maier, Thomas Längle, Jürgen Beyerer, Michael Heizmann

**Affiliations:** 1 Fraunhofer Institute of Optronics, System Technologies and Image Exploitation IOSB, 76131 Karlsruhe, Germanythomas.laengle@iosb.fraunhofer.de (T.L.);; 2 Institute of Industrial Information Technology (IIIT), Karlsruhe Institute of Technology, 76187 Karlsruhe, Germany; michael.heizmann@kit.edu

**Keywords:** sensor-based sorting, artificial intelligence, deep learning, transformer, CNN, real-time, bulky waste, food

## Abstract

**Highlights:**

**What are the main findings?**

- Transformer-based model achieves real-time performance in sensor-based sorting.
- Comparison of a CNN- and a transformer-based approach.
- U-Net shows faster inference and higher purity, whereas SegFormer achieves higher yield.

**What are the implications of the main findings?**

- The results indicate that, apart from CNNs, transformer-based architectures such as SegFormer, and potentially even vision–language models, are promising candidates for sensor-based sorting.

**Abstract:**

Conventional sensor-based sorting systems often rely on handcrafted image-processing features, which are highly task-specific, require expert knowledge, and are difficult to transfer to new sorting tasks. In contrast, deep learning enables automated feature learning and has previously been shown to outperform color-based sorting in several application scenarios. In this study, we investigate the suitability of two deep-learning-based semantic segmentation architectures, U-Net and SegFormer, for real-time sensor-based sorting. Their performance is evaluated in both offline experiments and practical sorting trials for the applications bulky waste sorting and hibiscus tea sorting. Both models proved suitable for the investigated tasks. U-Net showed slight advantages in inference speed and purity, reaching purities of 95.9 wt.% for bulky waste and 100.0 wt.% for hibiscus tea. SegFormer achieved almost equal results for sorting hibiscus tea and four percentage points lower purity for sorting bulky waste. Real-time applicability was demonstrated for SegFormer variants B0 to B3, which met the required throughput of 62 megapixels per second. Overall, the results show that transformer-based models are a viable and promising alternative to CNNs for real-time sensor-based sorting.

## 1. Introduction

Sensor-based sorting systems commonly rely on handcrafted image processing features. Those features struggle to capture complex visual patterns and perform reliably under dense material streams. While color cues are readily learnable and enable rapid system adaptation, more demanding classification tasks beyond simple color discrimination require manual definition of domain-specific features. This feature engineering process is highly material-dependent and must be re-performed by experts whenever the input material changes. Automated feature learning enabled by deep learning eliminates the need for hand-crafted feature engineering, thereby reducing the effort to adapt sorting systems to new materials. In addition, hand-crafted features are generally unable to match the sorting quality of deep learning approaches in sorting tasks that require the extraction of complex semantic information from images. Waste-stream sorting is one application domain that requires complex semantic information because waste streams contain a wide variety of materials, including composites. In a previous study, we demonstrated that deep-learning-based sorting can achieve significantly better results than color-based sorting. For sorting hibiscus tea, we improved accuracy by 15 percentage points and in construction and demolition waste by 20 percentage points [1].

Commonly used deep learning architectures in computer vision are Convolutional Neural Networks (CNNs) and Transformers. Transformers are often considered to be larger in terms of trainable parameters and have a higher computational demand compared to CNNs, which could be one reason that they are rarely deployed on an industrial sorting machine. As a matter of fact, transformers outperform CNNs on many computer vision tasks, including segmentation and object detection on benchmark datasets such as ADE20K, Cityscapes, and COCO [2,3,4]. Since the complexity of visual features within material streams for sorting applications varies, it remains uncertain under which conditions transformers are preferable.

In a previous work [1], we designed, implemented and evaluated an approach to sensor-based sorting using a CNN based on U-Net [5]. First, semantic segmentation is applied to the pre-processed input images to assign pixel-level class labels. The resulting segmentation masks are then mapped to the sorting system’s array of pneumatic valves. The developed pipeline is implemented for GPU-based execution and is capable of real-time processing. Implementation details of the training and inference pipeline are provided in [1].

In this study, we built upon the previous work by comparing the adapted U-Net to a transformer-based architecture called SegFormer [3] for sorting bulky waste and hibiscus tea.

## 2. Related Work

### 2.1. Deep Learning for Sensor-Based Sorting

Deep Learning has been increasingly applied to the optical characterization of material streams in sorting applications. In the waste management domain, this includes CNN-based classification of trash [6] and bulky waste using a multi-sensor approach [7] and, more recently, transformer-based classification approaches [8,9].

Segmentation of cluttered waste streams has also been addressed on real material from an industrial sorting facility [10]. In the food domain, CNN-based approaches have similarly been applied to quality inspection and defect detection, for example for peanuts [11,12]. More recently, transformer-based models have been adopted in this domain as well, including the grading of green coffee beans [13].

However, these studies evaluate detection or classification performance on recorded datasets and do not address deployment under the runtime constraints of an operating sorting machine. Studies that report physical sorting experiments with quantified separation results remain scarce.

### 2.2. Semantic Segmentation

Semantic segmentation assigns a class label to every pixel of an image and thereby provides the spatial precision and classification required for optical sorting machines. Semantic segmentation tasks have long been addressed by CNNs, beginning with Fully Convolutional Networks (FCNs) [14] and followed by architectures such as the DeepLab family [15], PSPNet [16] and HRNet [17]. A widely adopted representative is U-Net [5] combining an encoder–decoder structure with skip connections for precise localization. First introduced for biomedical imaging, it has proven effective in numerous domains, including sensor-based sorting [1].

Originally developed for natural language processing, the transformer architecture has also been applied to computer vision. The Vision Transformer (ViT) [18] demonstrated that a pure transformer operating on 16 × 16 image patches can match or surpass CNNs in image classification when trained at sufficient scale, capturing global context through the self-attention mechanism. Subsequently transformers were adopted for dense predictions with SETR [19] reformulating semantic segmentation as a sequence-to-sequence problem using a ViT backbone, achieving competitive accuracy but at higher computational cost, to be unsuitable for low-latency critical applications. Later transformer-based architectures, such as Mask2Former [20] further advanced accuracy at high computational cost, while others such as RTFormer [21] improved inference speed at the cost of accuracy. Balancing both aspects, SegFormer [3] combines a hierarchical Mix Transformer (MiT) with a lightweight all-MLP decoder. The encoder produces multi-scale features without requiring positional encodings, which renders the architecture robust to resolution changes between training and inference, a relevant property for sensor-based sorting, where camera setups and machine configurations may vary.

### 2.3. Real-Time Sorting

The work most closely related to this study is our previous investigation [1], in which a modified U-Net was employed for real-time semantic segmentation in sensor-based sorting and validated through physical sorting experiments. Other real-time deep-learning-based sorting systems have been demonstrated for agricultural grading [22,23]. Within the transformer family, Shen et al. [24] adopted the SegFormer for real-time contour extraction of aggregate particles in conveyor belt depth maps, yet without a downstream physical sorting system and evaluated only at recording speeds up to 0.26 m/s.

This work addresses these limitations by implementing and experimentally evaluating a transformer-based segmentation architecture on an industrial-grade sorting machine operating at a conveyor speed of 2.9 m/s. The transformer-based approach is compared against a CNN-based baseline in terms of sorting accuracy and inference speed.

## 3. Materials and Methods

### 3.1. Materials

We conducted the study using two sorting tasks. First, we consider the task of sorting bulky waste, with the objective of separating wood-related materials from non-wood items. The bulky waste samples were provided by the Fraunhofer Institute for Wood Research Wilhelm-Klauditz-Institut (WKI) in Braunschweig, Germany. The sample set consists of crushed furniture materials and non-wood-related materials, including plastic, metal, fabric, foam, and ceramic.

**Figure 1:**
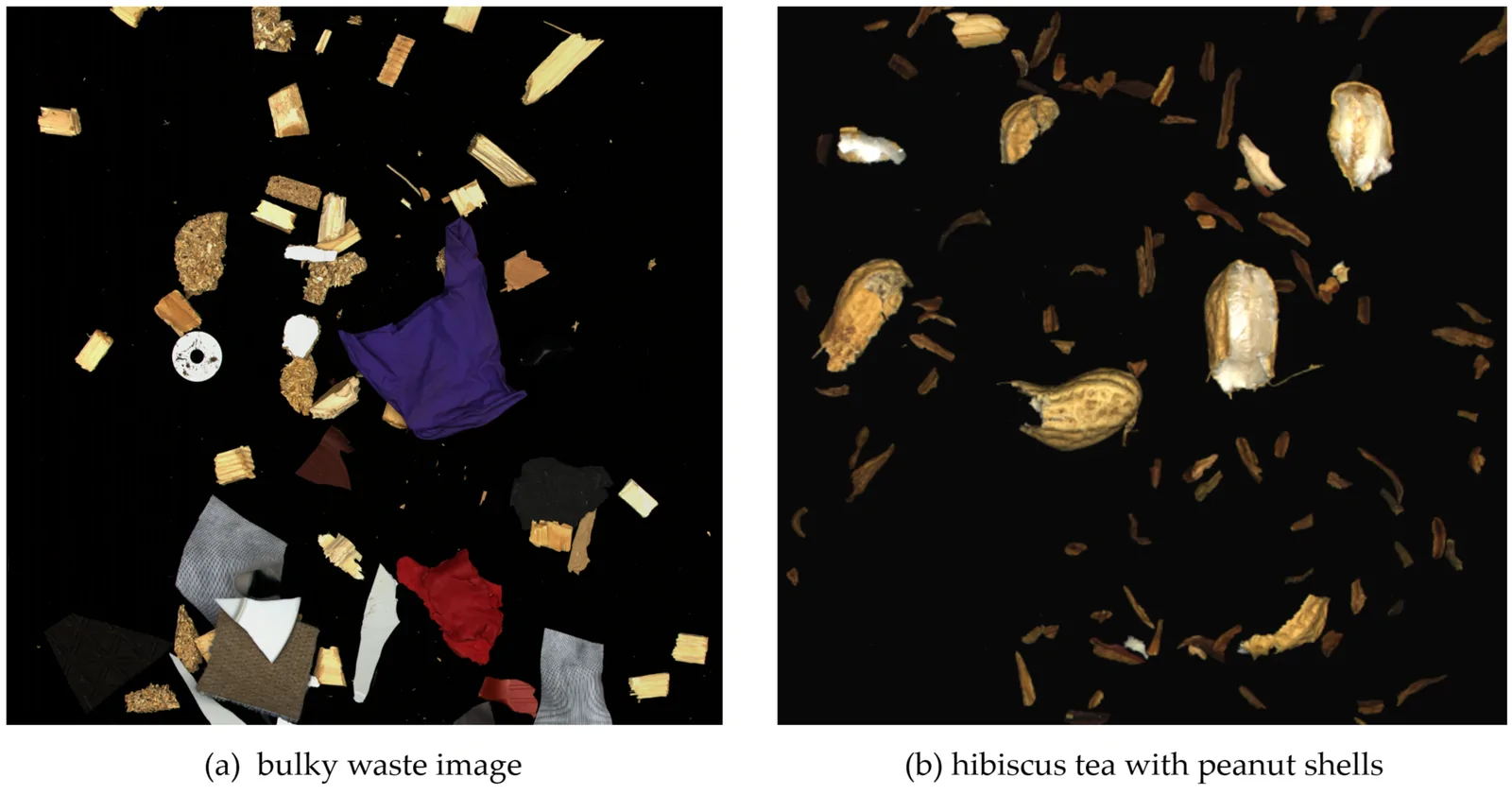
Materials used in the experiments. Sample set (**a**) consists of crushed furniture and non-wood-related materials, including plastic, metal, fabric, foam, and ceramic. Sample set (**b**) comprises hibiscus tea contaminated with peanut shells.

Second, we consider hibiscus tea, where peanut shells act as contaminants that must be removed from the material stream. Due to the similar color distributions of hibiscus and peanut shells, this sorting task presents a significant challenge for traditional color-based approaches. Example images for both tasks are illustrated in Figure 1.

### 3.2. Experimental Setup

**Figure 2:**
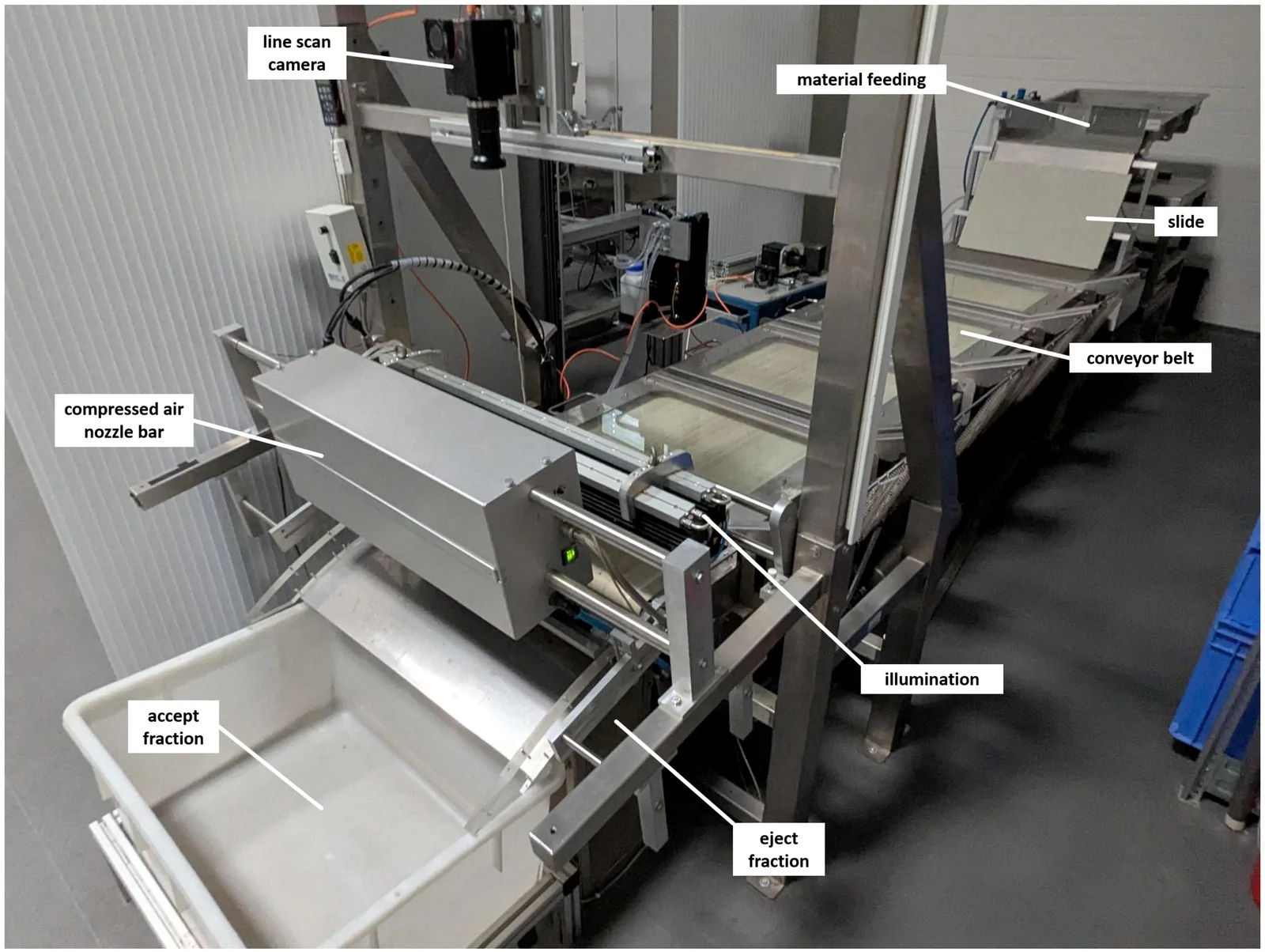
Sorting machine used for image data acquisition and sorting experiments. The setup features a conveyor belt width of 700 mm and a speed of 2.9 m/s, closely approximating typical industrial configurations.

For data acquisition and sorting experiments, we used the sorting machine illustrated in Figure 2. This laboratory-scale system was custom-built and closely resembles industrial configurations. 

Material is fed by a vibratory feeder supplied by VIBRA MASCHINENFABRIK SCHULTHEIS GmbH & Co., Offenbach am Main, Germany. Material is then accelerated via a slide onto a conveyor belt from HM Anlagentechnik GmbH, Stuhr‑Brinkum, Germany. The belt has a width of 700 mm and operates at 2.9 m/s. Pneumatic ejection is realized by a nozzle bar from HM Anlagentechnik and actuated by fast‑switching valves (MHJ9/10) from Festo SE & Co. KG, Esslingen am Neckar, Germany. The line scan camera is a prism‑based 4‑channel camera (SW‑4000Q‑10GE‑F) from JAI, Yokohama, Japan. Although the camera includes an additional near‑infrared channel, only the RGB channels were used in our experiments, so the system effectively operated as a standard RGB line scan camera. The line rate was set to 15,151 lines per second.

The inspection line is positioned directly at the edge of the conveyor belt to capture a clear black background rather than the belt surface. This configuration results in a distance of 120 mm between the inspection line and eject line, corresponding to approximately 40 ms between sensing and the ejection decision. The inspection line is illuminated by a high-power LED module with water cooling. Objects are ejected using a compressed air nozzle bar equipped with 128 nozzles.

### 3.3. Datasets

Using the described setup, we created datasets for bulky waste and hibiscus tea. We defined both as semantic segmentation tasks with three classes. Background, non-wood, and wood are the classes for the bulky waste dataset and background, peanut-shells, and hibiscus tea for the other dataset. By selecting two different application cases, we assessed the applicability of SegFormer and U-Net across diverse sorting scenarios. For each task, the models were trained and evaluated independently using task-specific data.

Training and validation data were generated synthetically by placing presorted objects on a background in random configurations, following the Cut-and-Paste concept [25]. This reduced manual labeling effort to the test data only. The random configurations included random translations, rotations, and scaling of the objects. Scaling factors were sampled uniformly between 0.9 and 1.1. No illumination variations were introduced. Occlusions of up to approximately 50% of an object’s pixel coverage were permitted. Only foreground pixels were overwritten through the alpha mask of the objects to be placed.

As very small objects are too time-consuming to place, they were omitted during data generation, which led to false detections of wood shavings in real test images. To address this, 20 recorded images of size 4096 × 4096 containing shavings were used as background for the synthetic bulky waste data. Such shavings were then consistently treated as background in the training, validation, and the test data. The example of wood shavings demonstrates a possible gap between synthetically generated training data and real test data. Investigating the extent of such gaps was not part of this work. However, minimizing these gaps would also be advantageous for real-world sorting tasks, as it would further reduce the need for manual labeling effort on real images.

The Bulky Waste dataset comprised 4096 synthetic training images and 512 synthetic validation images, all at 512 × 512 pixels, while testing was performed on 14 manually annotated real images of 4096 × 4096 pixels. The Hibiscus dataset consisted of 8192 synthetic training images and 512 synthetic validation images at 256 × 256 pixels, with 8 manually annotated real test images at 4096 × 4096 pixels. Although the number of test images may appear small, their high image size results in a substantial number of object instances in the test set. The exact number of object instances is reported in Table 1.

In an ablation study in Appendix B, we compare the effect of synthetic training data with that of real training data. We demonstrate that the synthetic data generation approach described here is a valid strategy for model training, achieving competitive performance compared with training on limited real data while requiring no manual, time-intensive annotation.

### 3.4. Methodology

In our previous work, we employed a modified lightweight U-Net [5] architecture for semantic segmentation. The modifications to the U-Net match [1]. The number of feature maps at each level is reduced by a factor of 4 compared to the original U-Net. Additionally, zero-padding is employed to ensure that the output dimensions match the input dimensions. This method yielded promising results, particularly for detecting peanut shells in hibiscus tea. However, large objects and those exhibiting complex textures still remain challenging. In some cases, larger objects showed inconsistent class labels across the object’s area. A reason for this might be the limited effective receptive field of the U-Net, a known drawback of CNNs pointed out by [3]. In the same study, they proposed the SegFormer, a transformer-based model for semantic segmentation. Instead of convolutional layers, transformer-based models leverage self-attention to capture long-range dependencies, resulting in larger effective receptive fields compared to CNNs [3]. In this study, we compare SegFormer to U-Net for the proposed sorting tasks.

The SegFormer architecture incorporates a Mix Transformer (MiT) encoder, which is inspired by Vision Transformer [18] but tailored for semantic segmentation. MiT is available in variants B0 to B5, ranging from 3.7 million to 84.7 million parameters. In contrast, the adapted U-Net used in this work contains only 1.96 million parameters.

We evaluate the models by implementing an end-to-end pipeline that spans RGB image acquisition to material ejection via compressed air nozzles. In semantic segmentation, each pixel is assigned to a material class, and these labels are mapped directly to valve actuation, thereby avoiding computationally expensive post-processing-one of the central features of our approach. In contrast, approaches based on object detection or instance segmentation require each detected object in the material stream to be processed individually. This leads to post-processing algorithms that are difficult to parallelize on GPUs and inference times that depend on the number of objects, making it more challenging to ensure compliance with the limited time window available for nozzle activation.

Our pipeline is compatible with any model that produces a semantic segmentation map. In Appendix C, we demonstrate this flexibility by comparing the introduced SegFormer and U-Net with additional architectures designed for fast inference under stringent real-time requirements.

### 3.5. Implementation Details

The SegFormer models were implemented in PyTorch (version 2.7.1+cu118) using the Hugging Face Transformers framework (version 4.52.4). We evaluated two training settings: fine-tuning from pretrained weights and training from scratch. For the pretrained setting, the models were initialized with ImageNet-1k-pretrained weights from the nvidia/mit-bX checkpoint, where X denotes the model variant. The models were subsequently fine-tuned for 10 epochs, requiring less than one hour. Training was stopped at this point because the loss no longer showed meaningful improvement. For training from scratch, the models were trained for 100 epochs, until the loss plateaued. This required substantially more time, taking up to 10 h. No additional data augmentation was applied apart from the synthetic data generation described in Section 3.3. Training used a batch size of 16 and the AdamW optimizer with a learning rate of 2 × 10^−4^ and a weight decay of 1 × 10^−5^.

The U-Net models were built in PyTorch (version 2.5.1+cu118) and trained from scratch, i.e., without pretraining. No additional augmentations were used beyond the synthetic data generation procedure described previously. Training was conducted with a batch size of 16 using the Adam optimizer with the default PyTorch settings (including a learning rate of 1 × 10^−3^). Depending on the experiment, training typically ran for 15 to 30 epochs, and the final model was selected based on the lowest validation loss.

After training, all models were exported to ONNX for efficient deployment in VisDevKit (VDK, version 1.2.13), a custom C++ framework developed at Fraunhofer IOSB for sensor-based sorting. This direct perception-to-actuation pipeline enables a simple, effective, and low-latency solution for real-time sorting applications. Model inference was performed on an NVIDIA RTX 5070 Ti, while training used an NVIDIA GeForce RTX 4090 for SegFormer and an NVIDIA GeForce RTX 3080 Ti for U-Net.

## 4. Results

### 4.1. Offline Results for Recorded Data

We first present the models’ performance on image data recorded with the sorting system’s camera. This serves as an important offline evaluation before conducting sorting experiments on the machine itself.

**Figure 3:**
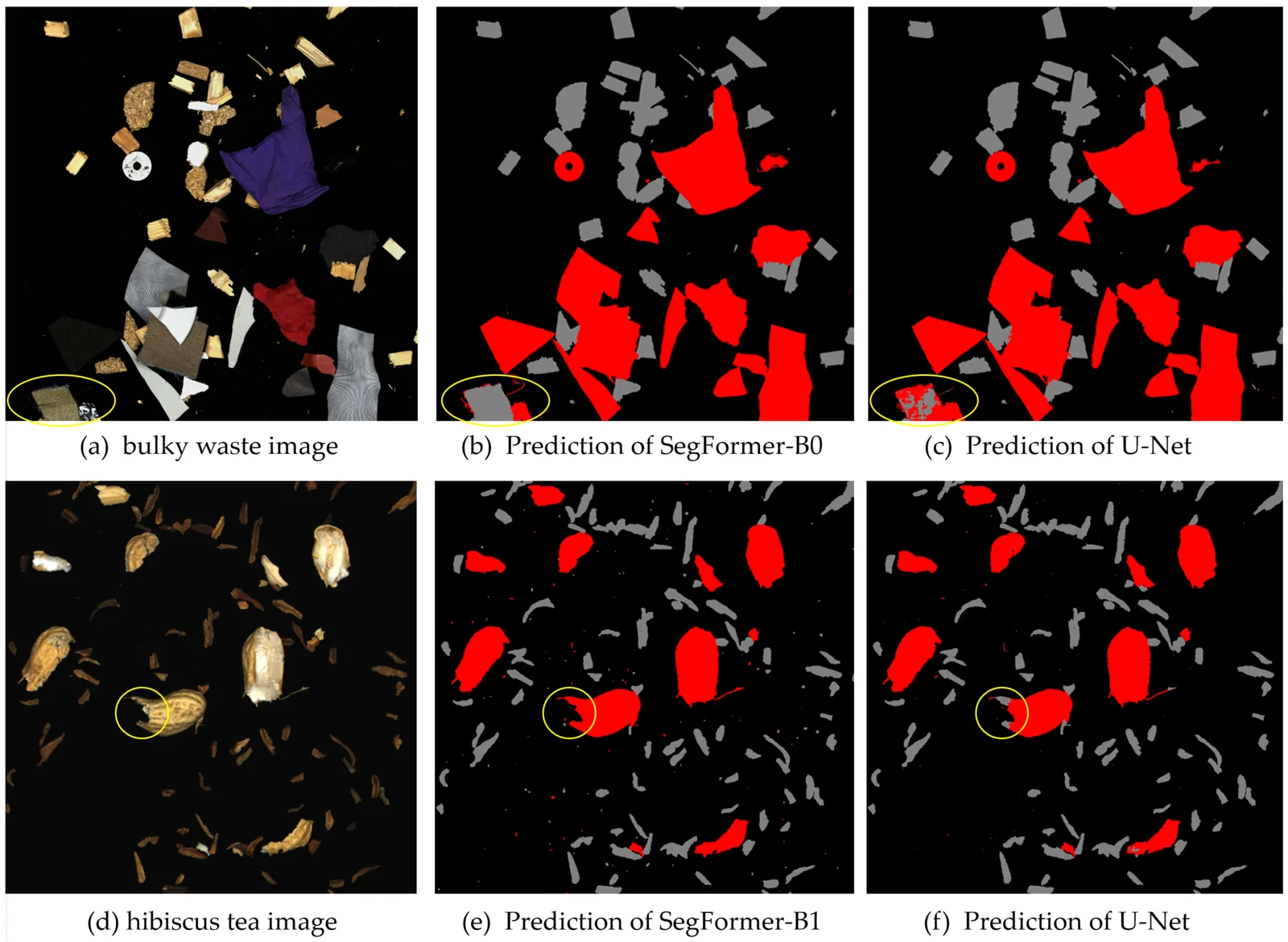
Qualitative results of the offline evaluation for the models SegFormer und U-Net. (**a**,**d**) show input images, whereas (**b**,**e**) present predictions of SegFormer and (**c**,**f**) predictions of U-Net. Regions to be ejected are red, regions identified as good material are gray, and the background is shown in black.

Figure 3 shows qualitative results of SegFormer and U-Net for both tasks. Panels (a) and (d) show the input images, whereas the other panels show the semantic segmentation predictions of the models. In semantic segmentation, one class is assigned to each pixel in the input image. The displayed images are crops from the test data used in the quantitative analysis. The regions highlighted by yellow circles illustrate some differences between the models. We present predictions generated by the pretrained SegFormer-B0 model for bulky waste and the pretrained SegFormer-B1 model for hibiscus tea. The same model configurations were selected for the subsequent sorting experiments, where the reasons for these choices are also explained.

As shown in Figure 3, SegFormer appears to produce more spatially consistent predictions, whereas U-Net tends to struggle with long-range context. As a result, the predicted class sometimes changes within a single object in the U-Net outputs, leading to less consistent predictions. Besides that, both models produced very pixel-precise predictions.

Using labeled test data, we further evaluated model performance quantitatively as presented in Table 2. Mean intersection over union (mIoU) was computed on a per-pixel basis for all classes, including the background. In contrast, the remaining metrics are instance-based and were obtained by counting correct and incorrect predictions for each instance. Instance (or object) regions were defined using manually annotated masks. Each instance was assigned a predicted class by majority voting over the predicted pixel classes within that instance. Note that this procedure was used for evaluation purposes only, since instance regions are not available during actual sorting. Nevertheless, it allows us to roughly estimate how many instances would have been sorted correctly if the corresponding input image had been observed.

We report several performance metrics including the four scores of a normalized confusion matrix: true positive rate (TPR), false positive rate (FPR), true negative rate (TNR), and false negative rate (FNR). For the interpretation of the results, we focus on two key metrics relevant to sorting tasks, which are yield (recall, true positive rate, TPR) and purity (precision, positive predictive value, PPV). We consider the non-eject class as positive and the eject class as negative. For example, the true negative rate (TNR) is defined as the number of correctly ejected instances divided by the total number of instances that should be ejected. In this experiment, these metrics are calculated on an instance basis by counting individual objects. Later, we will also report the corresponding scores on a weight basis, which is not possible here because this would require knowledge of the weight of each individual instance. We calculate the metrics for the modified U-Net as well as the SegFormer variants B0, B1, and B5. The SegFormer models were trained both from scratch and using the ImageNet-1k pretrained state published in study [3]. We summarize all results in Table 2.

On the bulky waste dataset, U-Net achieved the highest reported pixel-wise mIoU (95.60%) and purity (98.97%). Among the SegFormer models, SegFormer-B1 trained from scratch provided the most balanced performance, achieving the highest SegFormer mIoU (95.54%), instance accuracy (98.10%), and purity (98.72%). Compared with U-Net, SegFormer-B1 trained from scratch increased the yield from 98.46% to 98.97% (+0.51 percentage points), while its purity was 0.25 percentage points lower. Although SegFormer-B5 trained from scratch achieved the highest yield overall (99.49%), it obtained a substantially lower mIoU of 75.96% and a higher FPR of 13.10%. This indicates that its high recall was accompanied by poorer pixel-level segmentation quality and more false-positive detections.

On the hibiscus tea dataset, U-Net achieved the highest reported mIoU (94.65%), although its advantage over the pretrained SegFormer-B0 model (94.59%) was small. The pretrained SegFormer-B1 and SegFormer-B5 models achieved the highest purity (99.50%). SegFormer-B5 trained from scratch achieved the highest yield (96.77%) and instance accuracy (95.96%). However, similar as in the case of bulky waste, this model also produced the lowest mIoU (74.15%) and the highest FPR (10.85%).

Overall, increasing the SegFormer model size did not consistently improve performance. The smaller B0 and B1 variants matched or outperformed SegFormer-B5 in terms of mIoU, while SegFormer-B5 trained from scratch primarily favored high yield. The results also suggest that pre-training is important for stable pixel-level performance, particularly for the larger SegFormer variants. Thus, U-Net and SegFormer-B1 appear to provide the most balanced performance, whereas SegFormer-B5 trained from scratch may be suitable only in applications where maximizing yield is more important than minimizing false positives and achieving accurate segmentation boundaries.

### 4.2. Sorting Experiments

**Figure 4:**
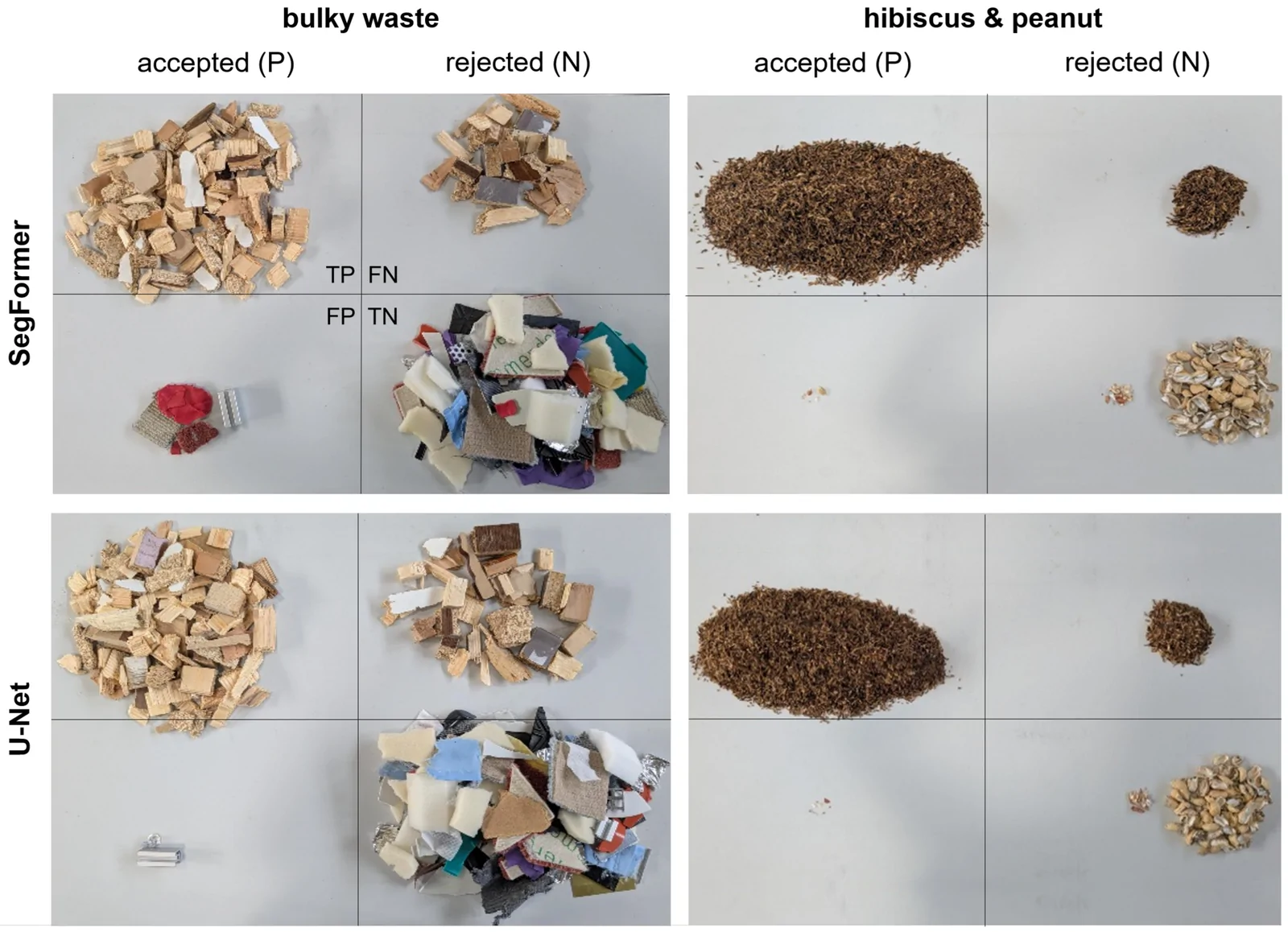
Qualitative sorting results of bulky waste and hibiscus tea with SegFormer and U-Net. Each image is split into four fractions: true positive (TP), false negative (FN), false positive (FP), and true negative (TN).

Besides the offline evaluation presented, we also conducted sorting experiments employing the models on a sorting machine, as described in Section 3.2. The two output streams of each sorting experiment are hand-sorted into four fractions as presented in Figure 4. Each image corresponds to the result of a single experiment. Based on the weight of each fraction, we report the same performance metrics as we have done for the offline evaluation.

#### 4.2.1. Sorting Bulky Waste

For sorting bulky waste, we perform experiments using pretrained SegFormer-B0 and U-Net. Pretrained SegFormer-B0 was selected as it achieved the best trade-off in terms of instance accuracy, training time, and inference speed. It outperformed all other pretrained SegFormer variants in the offline evaluation while also being the fastest architecture. As input stream, we use a mixture of 500 g wood and 500 g non-wood. We repeat each experiment three times. The qualitative results of the first experiment of SegFormer and U-Net are shown on the left side of Figure 4.

In Table 3 we report the quantitative sorting results for bulky waste and hibiscus tea. For the experiment of sorting bulky waste, we report mean values of the three runs with standard deviations in parentheses. It can be seen that the yield of SegFormer is almost 4 percentage points higher compared to U-Net, which means that there is less loss of wood. On the other hand, the purity is about the same number of percentage points lower, suggesting that SegFormer is accepting more material (wood and non-wood) in general compared to U-Net. This well-known tradeoff between precision and recall balances out when looking at the accuracy, being very similar for both models. Overall, both models achieved a good purity above 91%.

**Figure 5:**
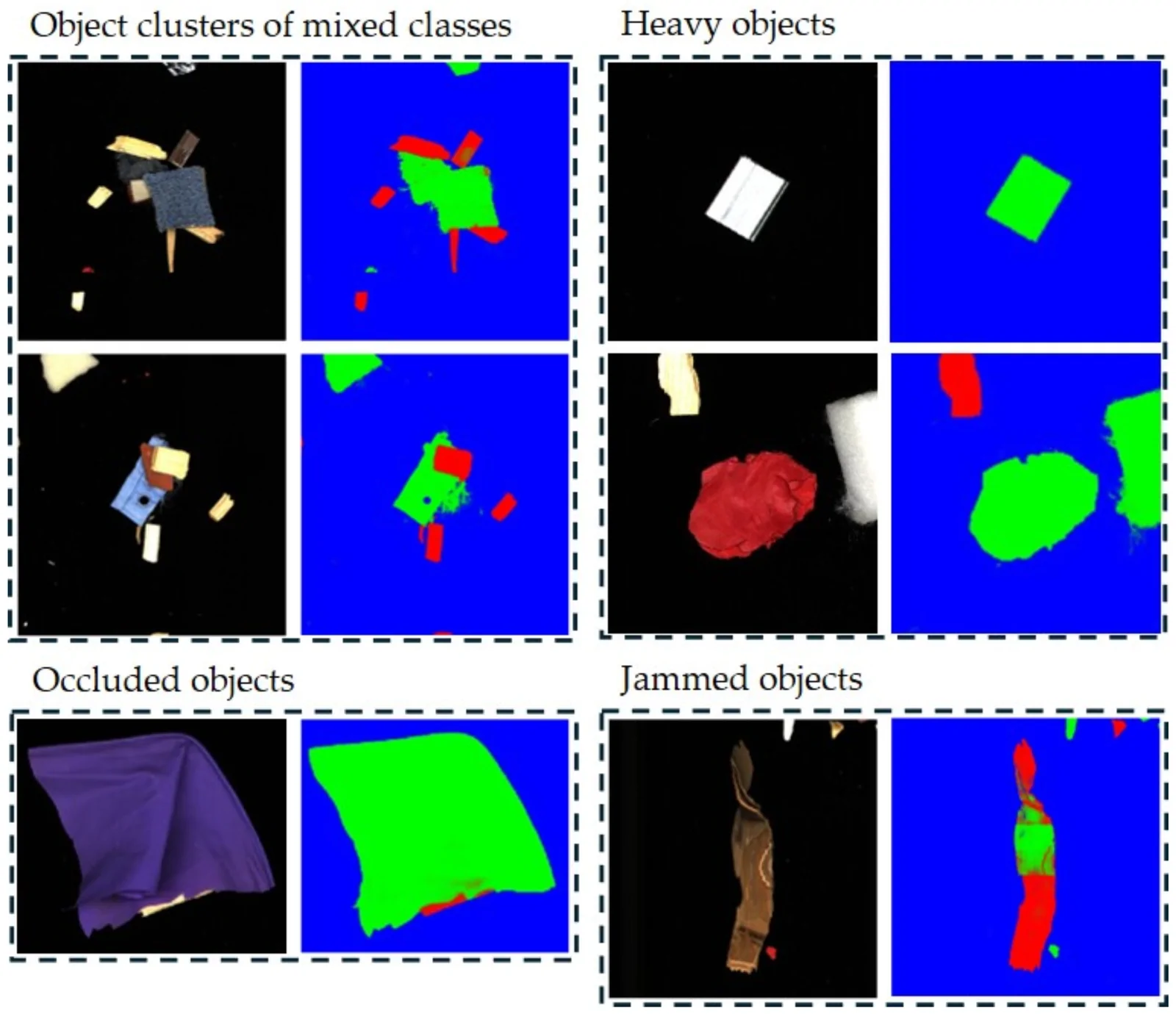
Qualitative analysis of error sources. For each error source (dashed lines), one or multiple examples are shown. The examples include a part of the input image to the model and the prediction of SegFormer. Predictions encode the output of the Softmax layer. Blue is background, green is reject, and red is accept. All shown examples were recorded during sorting.

The wood yield of 69.2% for SegFormer and 65.3% for U-Net indicates room for improvement. Compared with the offline evaluation results, which showed purities above 98% for bulky waste, this reveals a substantial performance gap. The discrepancy between detection and actual sorting suggests losses at the actuator stage or during the mechanical handling of the material. In fact, our setup was well suited for tea sorting, but bulky waste differs considerably in terms of object size and weight. This non-optimized setup likely explains part of the observed gap. Figure 5 depicts possible sources for the gap between online and offline experiments.

While the sensing stage provides high spatial precision, the ejection radius of the nozzles is comparatively large, causing neighboring objects to be ejected as well and thereby reducing the yield. This limitation becomes particularly pronounced when the proportion of material to be ejected is high, as the system must fire frequently, leading to the unintended removal of a substantial amount of correctly classified bulky waste. Figure 5 contains such object clusters where an activation of valves on non-wood material leads to the ejection of neighboring wood. This issue worsens when objects are mechanically interlocked, which is especially true for fibrous material. Some objects are also occluded. If the occluded object has a different material than the occluding object, the activation decision is fully driven by the occluding object. In offline experiments, occluded objects are not visible and therefore cannot be accounted in the metrics.

Figure 5 also depicts a selection of heavy objects that were encountered during sorting with SegFormer. The pictures were taken during the same sorting experiment, the results of which are depicted in Figure 4. While the model made a confident and correct decision, the valve activation was not strong enough to eject the material. Especially lightweight and large objects were observed to get stuck between the camera and the ejection line. An example is provided on the bottom right of Figure 5. This leads to elongated visual objects, which negatively impact prediction accuracy. Elongated objects indicate slower movement speeds, which decrease sorting accuracy due to the changed elapsed time between camera and valves.

These findings indicate key areas for future system improvements. Enhanced mechanical separation of objects could reduce the number and size of object clusters. Mechanical changes to the sorting system itself could prevent objects from getting stuck between the camera line and the pneumatic valves. Increasing the air pressure of ejection nozzles would allow for the reliable ejection of heavy objects. Implementing these system-level changes was beyond the scope of the present work, representing a significant limitation when interpreting the sorting results of bulky waste.

#### 4.2.2. Sorting Hibiscus Tea

For hibiscus tea sorting, we selected the pretrained SegFormer-B1 model and U-Net. SegFormer-B1 was chosen because it achieved the highest purity score in the offline evaluation while maintaining high yield and instance accuracy (Table 2). Although SegFormer-B5 trained from scratch achieved the highest instance accuracy, its inference speed was insufficient to meet the throughput requirements of our sorting machine. As input stream, we use a mixture of 400 g hibiscus and 20 g peanut shells. We perform each experiment one time and report the scores at the bottom of Table 3. Similarly, as in the case of sorting bulky waste, SegFormer is better in terms of yield, but this time only by 0.5 percentage points.

The purity of both models is 100.0% after rounding to one decimal place. When taking a look at Figure 4, it can be seen that there are some accepted peanut shells (FP), but their combined mass accounted for less than 0.1 percentage points and therefore did not affect the reported weight-based purity stated in Table 3. The corresponding FP objects are thin peanut skins, which are hard to distinguish from hibiscus tea and very light-weight. These peanut skins affect the instance-level metrics reported in Table 2 but have a negligible effect on the weight-based metrics reported in Table 3. This difference in metrics likely explains the higher purity values observed for the sorting experiments compared to the offline results. In weight-based purity, larger objects contribute more strongly, and these are often easier to classify because their larger size provides more contextual information than very small fragments.

In contrast to the use-case of sorting bulky waste, the proportion of material to be ejected can be assumed to be very low for sorting foreign materials from hibiscus tea. Because of that, the mixture is less than 20:1 in practice, shifting the actual yield from the measured TPR at a mixture of 20:1 to the yield for hibiscus only (mixture 1:0) being 99.0% for SegFormer and 99.2% for U-Net. As the hibiscus tea sorting experiment was conducted only once, we cannot report any standard deviation. Accordingly, the small observed difference in yield should not be regarded as statistically significant.

In summary, both models are very suitable for this task with a purity of almost 100% and a material loss of 1%, which can be achieved in the case of a low share of foreign material in the input stream (<5%). When comparing these results with the offline evaluation, we observe an improvement in both purity and yield for both models. This can be explained by the difference between the weight-based purity reported here and the instance-count-based purity used in the offline evaluation.

#### 4.2.3. Real-Time Capabilities of the Deep Learning Models

We tested the real-time capability of all models using the GPU NVIDIA GeForce RTX 5070 Ti. Inference is performed with TensorRT and Float16 precision. We process 256 accumulated lines using the models within VDK and report the inference latencies in Table 4. Additionally, the full GPU pipeline time is reported, being defined as the average duration for which the GPU is occupied per 256 image lines. Beyond model inference the GPU pipeline time contains pre- and postprocessing steps, copy operations to and from the GPU, and computational overhead.

The throughput requirement is determined by the camera frame rate, whereas the latency requirement is set by the maximum allowable time interval between sensing and nozzle activation in the sorting system. The minimum required throughput is therefore given by

$$\mathrm{throughput}\;\geq \;\mathrm{framerate}\;=\;\frac{1}{\mathrm{number}\;\mathrm{of}\;\mathrm{lines}\;\cdot \;\mathrm{line}\;\mathrm{cycle}\;\mathrm{time}}=\frac{1}{256\;\cdot \;66\;µs}=59.19\;s^{-1}\;.$$

The latency constraint is given by

$$\mathrm{latency}\;<\Delta t=\;\frac{\Delta s}{v}=\frac{120\;mm}{2.9\;ms^{-1}}=41.38\;ms,$$

where *Δt* denotes the time interval between sensing and nozzle activation, Δs is the distance between the sensing line and the nozzles, and v is the conveyor-belt velocity. The required throughput can also be expressed in megapixel per second, which is

$${\mathrm{throughput}}_{MP\;}\geq \;\frac{\mathrm{resolution}\;}{\mathrm{line}\;\mathrm{cycle}\;\mathrm{time}}=\;\;\frac{4096\;}{66\;µs}=\;62.06\;MP/s.$$

We measured the latency using timestamp-based measurements in the code. With that, we report the model inference time and the full GPU pipeline time in Table 4. Beyond model inference the full GPU pipeline time contains pre- and postprocessing steps, copy operations to and from the GPU, and computational overhead.

To determine the throughput, we gradually increased the frame rate until images were dropped due to processing-speed limitations. The throughput of U-Net, SegFormer-B0 and SegFormer-B1 exceeded the maximum frame rate that could be fed into the pipeline, which was 130.21 s^−1^. All evaluated models met the required image throughput, except for SegFormer-B4 and B5. B4 achieved a maximum framerate of 58.30 s^−1^ and B5 reached 52.79 s^−1^, which is below the required throughput of 59.19 s^−1^ (corresponding to 62.06 MP/s). Consequently, neither B4 nor B5 satisfies the real-time requirements of the sorting system.

From the frame rate, we further derived the number of processed pixels per second. Taking the inverse of the frame rate yields the mean processing time per image in terms of throughput, which can be compared with the full GPU pipeline time from the latency measurements. This comparison reveals that the throughput-derived processing time and the full GPU pipeline time are very similar in our case, indicating that the images are barely processed in parallel. Moreover, it becomes clear that the throughput requirement is the more restrictive constraint for the given sorting machine.

The results demonstrate the possibility of small- and medium-sized transformer-based models running in real time on a sorting system. Inference speed can be improved by downsampling the input image, though this may degrade model accuracy particularly when sorting small objects or when material-relevant information resides at fine spatial scales. A more cost-effective alternative is to utilize the token merging strategy from SegFormer++ [26], which reduces computational load while typically incurring a smaller accuracy trade-off.

We conducted an ablation study to assess the effects of downscaling and upscaling. For bulky waste, image resizing had little influence on the results, suggesting that the larger B4 and B5 variants may also be viable options. For example, downscaling the input by a factor of two reduces the number of pixels by a factor of four, lowering the required processing throughput to 15.51 MP/s. This would likely enable real-time operation of B4 and B5. Further details are provided in Appendix A.

## 5. Discussion

Overall, U-Net and SegFormer showed comparable performance in both the offline evaluation and the sorting experiments, indicating that both architectures are in principle well suited for the investigated tasks. Nevertheless, some differences became apparent when considering individual performance aspects. In particular, U-Net tended to outperform SegFormer in terms of inference speed and purity, which makes it an attractive option for real-time sorting applications where both throughput and sorting quality are critical.

The relative importance of purity and yield depends on the application scenario. In food processing, purity may take precedence, particularly when allergenic materials must be removed, as even minute impurities can substantially affect product quality and safety. Under such conditions, U-Net may be preferable because of its higher observed purity. However, whether this advantage over SegFormer extends to other domains remains to be established. Nevertheless, yield is still relevant in food processing, especially for high-value products. In recycling applications, the requirements for both purity and yield are often less stringent. Contamination may be difficult or impossible to avoid, for example when metal components remain attached to wooden parts, which can limit the achievable purity. In addition, the comparatively low value of many recycled materials generally makes lower yields more acceptable than in the food industry.

During our experiments we observed that training SegFormer from scratch was more likely to result in unstable training loss and poor convergence compared to using the pretrained variants. Specifically, convergence failed for SegFormer-B5 on the bulky waste dataset and for all SegFormer variants on the hibiscus tea dataset. While different training parameters might yield successful training from scratch, we did not investigate this further. From a practical standpoint, we recommend using the pretrained version of SegFormer. It consistently converged in approximately 10% of the training time required for training from scratch, while achieving almost equal performance.

Similar convergence problems appeared during training of U-Net, which was not pretrained. This suggests that the absence of pretraining may contribute to unstable training and that pretraining can help stabilize the optimization process. The SegFormer models used in this study were pretrained on ImageNet-1K, which comprises more than one million natural images. The corresponding pretrained weights are available in the official implementation released alongside the SegFormer paper [3].

We trained U-Net from scratch, which was motivated by practical constraints rather than by preferential reasons. In its original configuration, the U-Net architecture is not designed to handle three-channel inputs or to produce a full resolution semantic segmentation output and therefore had to be adapted to the task at hand. As a consequence of these architectural modifications, the original model weights cannot be used, and the network must be trained from scratch. Although it would in principle be possible to retain parts of the network and train only the modified components, but investigating this approach was beyond the scope of this study.

Compared to our previous study [1], the present results demonstrate that transformer-based architectures can also meet typical real-time requirements in sensor-based sorting, despite often being regarded as large and computationally demanding. This finding opens the door to the use of a broader range of transformer-based architectures, which are increasingly outperforming and replacing CNNs in many computer vision tasks. Leveraging vision–language models (VLMs), which are usually transformer-based, raises the prospect of sorting systems that could be guided by textual user input to support the segmentation and classification of materials, thereby enabling a degree of control and adaptation during deployment, even after training has been completed. A practical advantage of recently published high-impact architectures such as SegFormer is that they are available in multiple model sizes. This makes such models particularly attractive for sensor-based sorting, as they allow model selection based on the desired trade-off between predictive performance and inference speed.

## 6. Conclusions

In conclusion, both U-Net and SegFormer proved to be suitable for the investigated sensor-based sorting tasks, as they achieved comparable performance in both the offline evaluation and the sorting experiments. U-Net showed slight advantages in inference speed and purity, making it particularly attractive for real-time applications in which throughput and sorting quality are critical. Specifically, U-Net achieved a mean purity of 95.9 wt.% for bulky waste sorting, compared with 91.2 wt.% for SegFormer. In hibiscus tea sorting both models achieved an almost perfect purity with 100.0 wt.%. In contrast, U-Net showed lower material recovery with yields of 65.3 wt.% for bulky waste and 93.5 wt.% for hibiscus tea, compared to 69.2 wt.% and 94.0 wt.%, respectively, for SegFormer. The relative importance of purity and yield depends on the application. Purity may be prioritized in safety-critical food sorting, whereas moderate reductions in purity or yield can be more acceptable in recycling.

In comparison to our previous study [1], we showed that not only CNNs, but also transformer-based architectures can meet the real-time requirements of sensor-based sorting. Sufficient throughput was achieved with the SegFormer variants B0–B3 on a line-scan camera with 4096 pixels operating at 15,151 lines per second, corresponding to 62 megapixels per second, whereas larger models may require additional measures such as reducing the input resolution. The fastest models, U-Net and SegFormer-B0, were able to process the full GPU pipeline for a 256 × 4096 shaped image in 3.4 ms and 4.1 ms, respectively.

While only minor differences between U-Net and SegFormer were observed for the comparatively simple tasks studied here, more complex sorting scenarios may further increase the relevance of transformers. Overall, these findings demonstrate that transformer-based architectures are a viable and promising option for sensor-based sorting in real-time. This paves the way for the investigation of other transformer models for future sorting systems.

## Figures and Tables

**Table 1:** Number of object instances in the test data. The eject fraction is the fraction which is supposed to be ejected when it comes to sorting.

**Material**	**Non-Wood (Eject)**	**Wood (Non-Eject)**	**Total**
Bulky waste	84	390	474
**Material**	**peanut shells (eject)**	**hibiscus tea (non-eject)**	**Total**
Hibiscus tea	129	1085	1214

**Table 2:** Quantitative segmentation results of the models on offline data. B0 to B5 denote SegFormer variants, for which we report results using both pretrained backbones and training from scratch. Mean Intersection over Union (mIoU) is the only pixel-wise metric reported, whereas the remaining scores are instance-based metrics. The downward arrow (↓) indicates metrics for which lower values are better. For all other metrics, higher values are better. The best scores are highlighted in bold.

Material	Model	Pixel mIoU	Accuracy	PPV (Purity)	TPR (Yield)	FPR (↓)	TNR	FNR (↓)
Bulky waste	B0 pretrained	95.39%	96.62%	98.45%	97.44%	7.14%	92.86%	2.56%
	B1 pretrained	95.19%	95.36%	98.42%	95.90%	7.14%	92.86%	4.10%
	B5 pretrained	95.08%	95.99%	98.43%	96.67%	7.14%	92.86%	3.33%
	B0 scratch	95.52%	97.89%	98.47%	99.23%	7.14%	92.86%	0.77%
	B1 scratch	95.54%	**98.10%**	98.72%	98.97%	5.95%	94.05%	1.03%
	B5 scratch	75.96%	96.20%	97.24%	**99.49%**	13.10%	86.90%	**0.51%**
	U-Net	**95.6%**	97.89%	**98.97%**	98.46%	**4.76%**	**95.24%**	1.54%
Hibiscus tea	B0 pretrained	94.59%	92.67%	99.11%	92.63%	6.98%	93.02%	7.37%
	B1 pretrained	93.39%	92.50%	**99.50%**	92.07%	**3.88%**	**96.12%**	7.93%
	B5 pretrained	93.59%	92.50%	**99.50%**	92.44%	**3.88%**	**96.12%**	7.56%
	B0 scratch	93.56%	92.67%	99.11%	92.63%	6.98%	93.02%	7.37%
	B1 scratch	79.03%	92.59%	99.21%	92.44%	6.20%	93.80%	7.56%
	B5 scratch	74.15%	**95.96%**	98.68%	**96.77%**	10.85%	89.15%	**3.23%**
	U-Net	**94.65%**	93.00%	98.92%	93.18%	8.53%	91.47%	6.82%

**Table 3:** Quantitative sorting results of the models for both tasks. For bulky waste sorting, a 1:1 input material mixture by weight was used, whereas for hibiscus tea, a 20:1 mixture was applied. The last column reports the TPR for the case where only hibiscus tea was fed in the input (1:0 mixture). For bulky waste, values are reported as means with standard deviations in parentheses, given as percentage points. The downward arrow (↓) indicates metrics for which lower values are better. The best scores are highlighted in bold.

Material	Model	Accuracy	PPV (Purity)	TPR (Yield)	FPR (↓)	TNR	FNR (↓)	TPR for 1:0
Bulky waste	B0 pretrained	81.2% (0.4)	91.2% (2.7)	**69.2%** (1.6)	6.7% (2.4)	93.3% (2.4)	**30.8%** (1.6)	-
	U-Net	**81.4%** (1.8)	**95.9%** (2.5)	65.3% (2.0)	**2.7%** (1.7)	**97.3%** (1.7)	34.7% (2.0)	-
Hibiscus tea	B1 pretrained	**94.2%**	100.0%	**94.0%**	0.4%	99.7%	**6.1%**	99.0%
	U-Net	93.8%	**100.0%**	93.5%	**0.0%**	**100.0%**	6.5%	**99.2%**

**Table 4:** Latency and throughput for all models using an input image size of 1 × 256 × 4096 × 3 (batchsize × height × width × channels). Model sizes B4 and B5 were not able to achieve the required image throughput of 59.19 images per second.

	U-Net	SegFormer					
		B0	B1	B2	B3	B4	B5
**Latencies (ms)**							
Model inference	1.7 ms	2.5 ms	3.7 ms	9.3 ms	12.3 ms	15.2 ms	18.1 ms
Full GPU pipeline	3.4 ms	4.1 ms	5.3 ms	11.0 ms	14.0 ms	16.9 ms	19.7 ms
**Throughput**							
FPS	>130.21	>130.21	>130.21	95.27	73.70	58.30	52.79
Megapixel/s	>136.53	>136.53	>136.53	99.90	77.28	61.13	55.35
Full pipeline time (1/FPS)	<7.68 ms	<7.68 ms	<7.68 ms	10.50 ms	13.57 ms	17.15 ms	18.94 ms

## Data Availability

Data is available on request.

## Abbreviations

CNN	Convolutional Neural Network
ViT	Vision Transformer
MLP	Multilayer Perceptron
VDK	Vision Development Kit (software of Fraunhofer IOSB)
ONNX	Open Neural Network Exchange
FPS	Frames per Second
TPR	True Positive Rate
FPR	False Positive Rate
TNR	True Negative Rate
FNR	False Negative Rate
P	Positive
N	Negative
PPV	Positive Predictive Value
mIoU	mean Intersection over Union

## Appendix A

We investigated the effect of different input resolutions on SegFormer performance using the test datasets. The results are reported in Table A1 and were obtained using the pretrained SegFormer-B0 model for bulky waste and the pretrained SegFormer-B1 model for hibiscus tea.

**Table A1:** Quantitative segmentation results for different input scales. The results presented are from the pretrained SegFormer-B0 for bulky waste and pretrained SegFormer-B1 for hibiscus tea. The downward arrow (↓) indicates metrics for which lower values are better. For all other metrics, higher values are better. The best scores are highlighted in bold.

Material	Scale	Pixel mIoU	Accuracy	PPV (Purity)	TPR (Yield)	FPR (↓)	TNR	FNR (↓)
Bulky waste	0.25	94.01%	96.20%	98.19%	97.18%	8.33%	91.67%	2.82%
	0.5	94.08%	**96.84%**	**98.45%**	**97.69%**	**7.14%**	**92.86%**	**2.31%**
	1	**95.39%**	96.62%	**98.45%**	97.44%	**7.14%**	**92.86%**	2.56%
	1.5	93.97%	96.20%	98.44%	96.92%	**7.14%**	**92.86%**	3.08%
Hibiscus tea	0.25	83.44%	82.70%	99.00%	81.75%	6.98%	93.02%	18.25%
	0.5	91.49%	92.34%	98.82%	**92.53%**	9.30%	90.70%	**7.47%**
	1	**93.39%**	**92.50%**	**99.50%**	92.07%	**3.88%**	**96.12%**	7.93%
	1.5	92.30%	92.26%	99.21%	92.07%	6.20%	93.80%	7.93%

For bulky waste, performance was less sensitive to input scaling than expected. In particular, downscaling the input images to a scale factor of 0.25 did not substantially degrade segmentation performance. One possible explanation is that objects in bulky waste images are generally large, making fine spatial details less critical for accurate segmentation. In contrast, downscaling hibiscus tea images to a scale factor of 0.25 resulted in a considerable performance decrease, with mIoU and accuracy declining by almost 10 percentage points. Purity, however, was only marginally affected. Overall, downscaling by a factor of two, corresponding to a scale factor of 0.5, generally achieved better results than upscaling to a scale factor of 1.5 and, in some cases, even outperformed evaluation at the original resolution.

Downscaling the input dimensions by a factor of two reduces the number of pixels by a factor of four and can therefore substantially decrease the computational requirements, most likely enabling the use of larger SegFormer variants B4 and B5. However, this finding may not generalize to applications involving very small objects or tasks that depend on fine-grained visual details.

## Appendix B

To evaluate the impact of synthetic training data, we compared three training settings: training exclusively on synthetic data, training exclusively on real data, and mixed training using first synthetic and then real data. A pretrained SegFormer served as the initial model in all experiments. For this purpose, images were recorded using the sorting machine setup as described in Section 3.2 and subsequently annotated. The images were divided into patches of 256 × 256 pixels, resulting in 577 training and 64 validation patches. In the mixed-data setting, the model was first fine-tuned on the synthetic dataset described in Section 3.3 and subsequently fine-tuned on the real dataset. The results obtained with real-only and mixed training were compared with those previously achieved by fine-tuning the pretrained SegFormer exclusively on synthetic data. The quantitative results are summarized in Table A2.

**Table A2:** Quantitative segmentation results using real and mixed training data of hibiscus tea. A pretrained SegFormer was used as the initial model in all experiments. We compare the model performance to the previously obtained results of synthetic fine-tuning (synth). The downward arrow (↓) indicates metrics for which lower values are better. For all other metrics, higher values are better. The best scores are highlighted in bold.

Data	Model	Pixel mIoU	Accuracy	PPV (Purity)	TPR (Yield)	FPR (↓)	TNR	FNR (↓)
synth	B0 pretrained	**94.59%**	92.67%	99.11%	92.63%	6.98%	93.02%	7.37%
	B1 pretrained	93.39%	92.50%	**99.50%**	92.07%	**3.88%**	**96.12%**	7.93%
	B5 pretrained	93.59%	92.50%	**99.50%**	92.44%	**3.88%**	**96.12%**	7.56%
	B0 pretrained	93.23%	94.98%	97.76%	96.59%	18.60%	81.40%	3.41%
real	B1 pretrained	93.25%	94.48%	97.57%	96.22%	20.16%	79.84%	3.78%
	B5 pretrained	93.91%	95.39%	97.86%	96.96%	17.83%	82.17%	3.04%
	B0 pretrained	93.65%	95.30%	97.42%	97.33%	21.71%	78.29%	2.67%
mixed	B1 pretrained	94.01%	94.89%	98.03%	96.22%	16.28%	83.72%	3.78%
	B5 pretrained	93.79%	**95.47%**	97.51%	**97.42%**	20.93%	79.07%	**2.58%**

Overall, training on synthetic data achieved competitive performance. In particular, the synthetic-only setting obtained the highest pixel-level mIoU, purity, and TNR, as well as the lowest FPR. However, for SegFormer-B5, mixed training achieved the highest accuracy and yield, together with the lowest FNR. The differences between the training settings were generally small, and fine-tuning exclusively on real images achieved performance comparable to that of the other approaches. Although it may initially seem counterintuitive that training on real data performed slightly worse than training on synthetic data, the synthetic dataset contained 8192 samples, compared with only 577 samples in the real training set. Therefore, the larger size of the synthetic dataset may explain its superior performance.

These results indicate that the synthetic data generation approach described in Section 3.3 is a valid strategy for model training, demonstrating that it can achieve competitive performance compared with training on limited real data. The synthetic data generation enables the creation of a labeled dataset from images of presorted fractions without requiring manual, time-intensive annotation.

## Appendix C

In this section we compare the investigated models with additional deep-learning architectures designed for fast inference under stringent real-time requirements. We include only models for semantic segmentation that could be readily integrated into the processing pipeline described in Section 3.4. Specifically, we evaluated DeepLabv3+ [15] and a Feature Pyramid Network (FPN) [27], two widely used CNN-based architectures, as well as LinkNet [28], which was developed for efficient semantic segmentation. Each architecture was combined with an encoder backbone. We selected ResNet-18 [29] as a high-performing yet relatively shallow backbone and MobileNetV2 [30], because it incorporates computationally efficient convolutional layers. To further investigate the influence of backbone selection, we also evaluated U-Net variants with the same backbones. Table A3 summarizes the segmentation results for all evaluated models.

**Table A3:** Quantitative segmentation results for various baseline models. All models are trained from scratch, except for SegFormer-B1. The downward arrow (↓) indicates metrics for which lower values are better. For all other metrics, higher values are better. The best scores are highlighted in bold.

Model	Backbone	Pixel mIoU	Accuracy	PPV (Purity)	TPR (Yield)	FPR (↓)	TNR	FNR (↓)
LinkNet	Resnet18	88.21%	92.26%	**99.60%**	91.71%	**3.10%**	**96.90%**	8.29%
U-Net	Resnet18	94.30%	93.33%	99.51%	93.00%	3.88%	96.12%	7.00%
U-Net	MobileNetV2	90.90%	92.59%	99.31%	92.35%	5.43%	94.57%	7.65%
FPN	MobileNetV2	90.10%	93.57%	99.03%	93.73%	7.75%	92.25%	6.27%
DeepLabv3+	MobileNetV2	92.20%	**93.66%**	99.03%	**93.82%**	7.75%	92.25%	**6.18%**
B1 pretrained	MiT	93.39%	92.50%	99.50%	92.07%	3.88%	96.12%	7.93%
U-Net	-	**94.65%**	93.00%	98.92%	93.18%	8.53%	91.47%	6.82%

DeepLabv3+ achieved the highest accuracy and yield, whereas LinkNet achieved the highest purity. Our custom U-Net attained the highest pixel mIoU. Although no single model achieved the best score for every metric, DeepLabv3+ provided a favorable overall trade-off, particularly for the instance-based metrics. The custom U-Net performed comparably to the U-Net with a ResNet-18 backbone and achieved a higher pixel mIoU. In contrast, replacing ResNet-18 with MobileNetV2 reduced the performance of the U-Net for every reported segmentation metric. These results suggest that ResNet-18 is better suited than MobileNetV2 for the hibiscus tea sorting task.

Table A4 presents the latency measurements for the additionally evaluated models. The full GPU-pipeline latency ranged from 2.9 ms to 4.9 ms. LinkNet achieved the lowest latency, with a full GPU-pipeline time of 2.9 ms, whereas pretrained SegFormer-B1 exhibited the highest latency among the tested models. Interestingly, ResNet-18 was marginally faster than MobileNetV2 in the U-Net comparison, reducing the full GPU-pipeline latency by 0.1 ms. LinkNet combined the lowest full GPU-pipeline latency with the highest purity. However, it also showed the lowest pixel mIoU of all models in Table A3, reaching 88.21%. If pixel mIoU is the primary criterion, the custom U-Net may be the preferable choice, as it achieved a pixel mIoU of 94.65% with a full GPU-pipeline latency of 3.1 ms, only 0.2 ms slower than LinkNet.

**Table A4:** Latencies measured for various baseline models using an input image size of 1 × 256 × 4096 × 3 (batchsize × height × width × channels). The best scores are highlighted in bold.

Model	Backbone	Latency	
		Model Inference	Full GPU Pipeline
LinkNet	Resnet18	**1.5 ms**	**2.9 ms**
U-Net	Resnet18	1.9 ms	3.3 ms
U-Net	MobileNetV2	2.0 ms	3.4 ms
FPN	MobileNetV2	1.8 ms	3.2 ms
DeepLabv3+	MobileNetV2	2.0 ms	3.4 ms
B1 pretrained	MiT	3.5 ms	4.9 ms
U-Net	-	1.6 ms	3.1 ms

We do not report throughput values because all models in Table A4 were able to process more than 130.21 frames per second, which corresponds to the maximum image-stream rate that the camera was able to deliver. The ablation study of this section was conducted several months after the original experiments in Section 4.2.3. Therefore, U-Net and SegFormer B1 latencies were measured again to ensure identical system settings. The discrepancy between these measurements and those reported in Section 4.2.3 is likely due to different operating system or driver versions, since no software or configuration changes were performed.

The additional experiments demonstrate the flexibility of the described approach, as the model used is easily interchangeable. For real-world sorting, the developer could select any of the presented models based on the relevant performance metrics for the specific use case, since all models exhibited similar real-time capabilities.
